# Parity-time-symmetry enhanced optomechanically-induced-transparency

**DOI:** 10.1038/srep31095

**Published:** 2016-08-04

**Authors:** Wenlin Li, Yunfeng Jiang, Chong Li, Heshan Song

**Affiliations:** 1 School of Physics and Optoelectronic Engineering, Dalian University of Technology, Dalian 116024, China; 2 Materials Science and Engineering, University of California, San Diego, 9500 Gilman Drive, La Jolla, CA 92093-0418, USA

## Abstract

We propose and analyze a scheme to enhance optomechanically-induced-transparency (OMIT) based on parity-time-symmetric optomechanical system. Our results predict that an OMIT window which does not exist originally can appear in weak optomechanical coupling and driving system via coupling an auxiliary active cavity with optical gain. This phenomenon is quite different from these reported in previous works in which the gain is considered just to damage OMIT phenomenon even leads to electromagnetically induced absorption or inverted-OMIT. Such enhanced OMIT effects are ascribed to the additional gain which can increase photon number in cavity without reducing effective decay. We also discuss the scheme feasibility by analyzing recent experiment parameters. Our work provide a promising platform for the coherent manipulation and slow light operation, which has potential applications for quantum information processing and quantum optical device.

In quantum mechanics and quantum optics, it seems almost inevitable that the quantum system will interact with the environment around it. The dynamics of such an open quantum system can be described by a non-Hermitian Hamiltonian (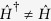) with complex eigenvalues, which will result in different phenomena from ones in closed quantum system^1^. Recently, a special class of physical system with a so-called parity-time (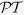) symmetry has attracted great attention^2^,^3^,^4^,^5^,^6^,^7^,^8^,^9^. It has been proved that a 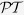-symmetric non-Hermitian Hamiltonian (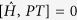) can also have real eigenvalue spectra^10^,^11^. This characteristic make open system subject to unitary time evolution like closed system, which causes some environmental damage effects, for example decoherence, can be suppressed effectively. Up to now, 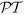-symmetric system has shown its extensive application prospects in quantum optics and quantum information processing (QIP), including strengthening optics nonlinearity^12^,^13^, enhancing photon blockade^14^, realizing quantum chaos^15^, soliton active controlling^16^,^17^, and so on.

In addition to theoretical research, 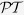-symmetric Hamiltonian has also been realized experimentally in a variety of physical systems^18^,^19^,^20^,^21^,^22^. Among them, a simple and intuitive scheme is to link two coupled cavities with optical gain and loss respectively. Especially in refs 18,20, 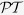-symmetric region is obviously observed by measuring the eigenfrequencies of supermodes. These progresses of experiments provide a solid platform for exploring the fundamental 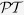 symmetry and corresponding strange phenomena.

Although (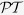)-symmetry can enhance many quantum optical effects by balancing the loss with extra gain, there still are opposite phenomena in which environmental loss play a positive role. The most typical two of such phenomena are optical cooling^23^,^24^,^25^ and electromagnetically induced transparency (EIT)^26^,^27^,^28^. It is well known that a larger decay rate is necessary whether for EIT in atomic system^29^ or for optomechanically-induced-transparency (OMIT) in cavity optomechanical system (OMS)^26^, and a gain is considered just to damage EIT (OMIT) phenomenon even leads to electromagnetically induced absorption (EIA) or inverted-EIT (inverted-OMIT) in previous works^30^,^31^. Up until now, enhancing EIT or OMIT still relies on additional coupled dissipation systems^32^,^33^,^34^,^35^. Correspondingly, the extra dissipation needs strong driving and nonlinear coupling to maintain the interference effect in OMIT, which still remains twofold difficulties because strong driving may lead nonlinear system to instability^15^ and strong nonlinear coupling is hard to realize in OMS^18^.

In this paper, we propose and analyze an enhanced optomechanically induced transparency in OMS via coupling an auxiliary active cavity with optical gain. A transparency window can appear in weak optomechanical coupling and driving system, however, similar window can not be obtained in dissipation system unless the driving (or the optomechanical coupling) intensity is magnified roughly 20000 times. This scheme is quite different from ones in existing works because our results show that extra gain can exert positive effect on OMIT phenomenon. Though the conclusion is counter-intuitive, we have analyzed the physical mechanisms of our scheme and explained the reasons of this kind of enhancement. After simulating, we find that absorption and transparency can be of controllable flexibilities by switching the fiber. We believe it can provide a promising platform for the coherent manipulation and slow light operation.

## Results

In this part, we present the main results of this work by introducing dynamics analyses and enhanced electromagnetically induced transparency phenomena in parity-time-symmetric optomechanical system. The details of calculation and simulation can be found in METHODS.

### Model and dynamics analysis

**Figure 1:**
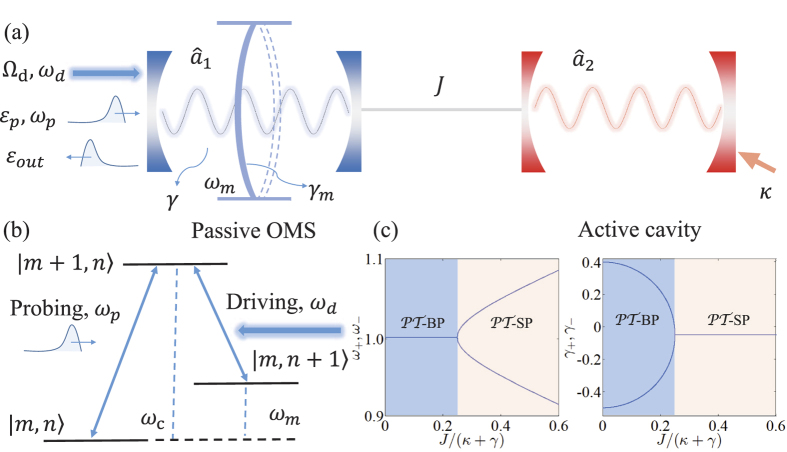
(**a**) Schematic illustration of our enhanced OMIT. Here the 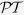-symmetric OMS consists of a passive OMS coupled to an active cavity with tunnelling strength *J*. (**b**) Level diagram of the OMIT in OMS. |*m*, *n*〉 denotes the state of *m* photons and *n* phonons in the displaced frame. (**c**) Eigenfrequencies of supermodes as a function of coupling *J*/(*κ* + *γ*).

We consider a typical 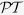-symmetric system for realizing the enhanced OMIT effect (see Fig. 1 for detail) and it is necessary to analyze its dynamics firstly.

By setting 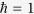, the total Hamiltonian of such a system can be written by increasing oscillator free term and optomechanical interaction on the basis of ordinary cavity Hamiltonian^31^, i.e.,


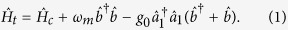


The Hamiltonian of the cavity can be written as 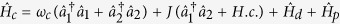, where 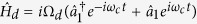 denotes the Hamiltonian of the driving field and correspondingly, the the probe field inside the cavity is described by 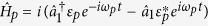. Here we make a frame rotating via 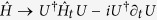 and let 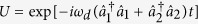, then the total Hamiltonian of the whole system becomes:


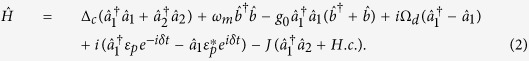


In this expression, 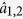 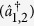 and 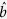 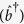 are the annihilation (creation) operators of the cavity mode and mechanical mode, respectively. Ω_*d*_ and *ε*_*p*_ are the amplitudes of the driving field and the probe field, which are respectively related to the input powers (*P*_*d*_ and *P*_*p*_) and decay rate (*γ*), i.e., 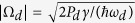 and 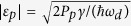. 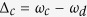 is the detuning between the driving field and cavity field. *γ* (*γ*_*m*_) is the optical (mechanical) decay rate and *κ* is the gain rate of the active cavity. *g*_0_ is the single-photon coupling intensity of the radiation pressure interaction between the passive field and the oscillator. For the convenience of discussion, we define the dimensionless position operator 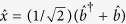 and momentum operator 
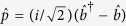
 instead of the phonon operators in Eq. (2). Then the dynamics of such a system can be determined by quantum Langevin equation 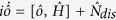, where 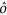 is an arbitrary optical or mechanical operator and 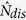 is the dissipation term.

Note that both the stability analyses in 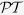-symmetric system and the OMIT phenomena in OMS need to be considered only in the mean value regime^15^,^26^. Similarly to previous EIT works in atomic systems, optomechanically induced transparency only deals with the mean response of the system to the probe field without including quantum fluctuation. On the other hand, the non-Hermitian Hamiltonian will be regard as an exact description of an OMS only if the input-bath operators are ignored. Therefore in order to explore the nonlinear dynamics of the system, we employ the semi-classical Langevin equations of motion, that is, set 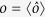 in the corresponding quantum Langevin equations. Then the mean values can be obtained by following dynamics equations^15^,^36^:


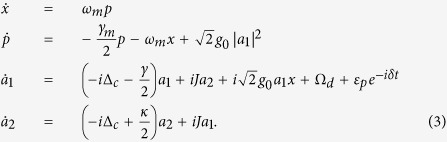


A necessary condition for obtaining an available OMIT phenomenon is that there should exist an asymptotic steady state and the corresponding system will keep the state for a long evolution time. As we all know, the probe field in OMIT typically has a small amplitude and it would not have an influence on the stability of the system. Therefore, here we firstly ignore the term 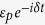 in Eq. (3) for a more simple stability analysis and the stability or stochastic property of the system is characterized by the Jacobian matrix


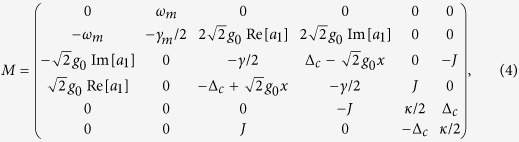


which can be obtained by linearizing Eq. (3) under the base vector 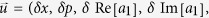

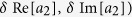
. Here we neglect the optomechanical interaction in Eq. (4) under the condition of weak-coupling regime 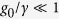. In this case, the eigenvalues of this Jacobian matrix can be easily solved as


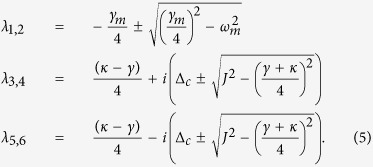


The real parts of above eigenvalues are also known as the Lyapunov exponents of this nonlinear dynamical system^37^,^38^,^39^. Whether the system is stable or not can be judged by comparing the largest Lyapunov exponent with zero^38^,^40^. Equation (5) implies that if *J* > (*κ* + *γ*)/4, the second terms in *λ*_3,4,5,6_ will be pure imaginary numbers. The real parts of these eigenvalues will not change with the parameter *J* and the corresponding Lyapunov exponents are *L*_*y*1,2_ = −*γ*_*m*_/4 and *L*_*γ*3,4,5,6_ = (*κ* − *γ*)/4. If the decay and gain rates are in the regime *κ* < *γ*, all Lyapunov exponents of this system will be negative, which can ensure that the mean value trajectories will tend to a fixed point in phase space. On the contrary, the second terms in *λ*_3,4,5,6_ will present non-zero real parts if *J* < (*κ* + *γ*)/4, which causes positive Lyapunov exponents. In the unstable system, neglected terms *g*_0_Re[*a*_1_], *g*_0_Im[*a*_1_] and *g*_0_*x* in Jacobian matrix will continue to be amplified, and this phenomenon is equivalent to enlarging the nonlinear coupling coefficient and the system will become more unstable in this case.

**Figure 2:**
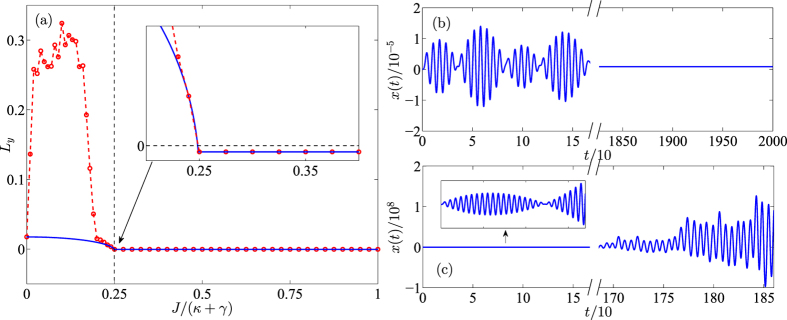
(**a**) Analytical (blue) and numerical (red) Lyapunov exponents as a function of coupling *J*/(*κ* + *γ*). (**b**,**c**) Evolutions of dimensionless mechanical displacement *x* in 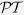-SP and 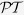-BP, respectively. The dimensionless parameters in this simulation are: Δ_*c*_ = *ω*_*m*_ = 1, *γ* = 1/23.4 = 0.043, *γ*_*m*_ = 0.038*γ* = 1.652 × 10^−3^, *g*_0_ = 7.4 × 10^−5^*γ* = 3.217 × 10^−6^, 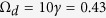 and 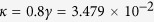. In (**b**,**c**) *J*/(*γ* + *κ*) is taken as 1 and 0.2, respectively.

Here we give the feasibility analysis of our parameters used in above discussions. The dimensionless parameters are in accord with recent microcavity experiments, that is, *ω*_*c*_ = 190 THz and *ω*_*m*_/2*π* = 23.4 MHz. The *Q*-factor of passive cavity is 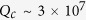 corresponding to *γ* ~ 6.33 MHz and *γ*/2*π* ~ 1 MHz. The optomechanical interaction intensity in this cavity is *g*_0_ = 7.4 × 10^−5^*γ* and corresponding oscillator decay is *γ*_*m*_/2*π* ~ 38 kHz owing to *Q*_*m*_ ~ 2*Q*_*c*_/10^5 ^18,19,20. The driving and probe powers are *P*_*d*_ ~ 8 pW and *P*_*p*_ ~ 0.08 pW, respectively. In addition, a small gain rate and fiber coupling (*J*, *κ* < *γ*) are also easy to be achieved in experiments^41^,^42^. After adopting these parameters, we select *ω*_*m*_ = 1 as an unit to nondimensionalize the other parameters, i.e., *γ* = 1/23.4, *γ*_*m*_ = 0.038*γ* and Ω_*d*_ = 10*γ*. It can been found that the parameters used in our simulations are similar with ones in previous works^15^,^30^. In Fig. 2, we verify above discussions by numerically calculating the oscillator evolutions and the largest Lyapunov exponents. Figure 2(a) shows that the critical point of the system stability is at *J* = (*κ* + *γ*)/4. When *J* is increased to be greater than the critical point, a significant asymptotic steady state emerges in this system (see Fig. 2(b)). On the other hand, decreasing *J* pushes the system to chaotic motion (see Fig. 2(c)). Since the nonlinear term is enlarged in this case, there exists a deviation for the largest Lyapunov exponents between the numerical solution and the corresponding analytical solution. However, the analytical largest Lyapunov exponent is accurate when the system is stable.

After ignoring the oscillator dissipation, we can make an inversion based on Eq. (3) and obtain following non-Hermitian Hamiltonian


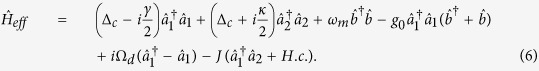


to describe this 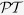-symmetric system. By diagonalizing the non-Hermitian Hamiltonian, one can easily find that *J* = (*κ* + *γ*)/4 is also the exceptional point (EP) of the transition from the 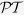-symmetric phase (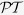-SP) to 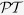 -symmetry breaking phase (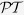-BP). Therefore, we emphasize such a conclusion that the system will always be stable in 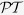-SP. This is the essential prerequisite for enhancing OMIT by using 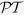-symmetric OMS.

### Enhanced optomechanically induced transparency

The steady-state solution of Eq. (3) can be expanded to contain many Fourier components. In the limit of weak probe field, the high order terms of *ε*_*p*_ are neglected in our work and each operator will have the following form^26^,^43^:


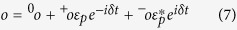


under the condition of *t* → ∞. Substituting Eq. (7) into Eq. (3) and ignoring these terms containing 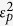, 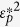 and |*ε*_*p*_|^2^, we can respectively gain the zero-order and first-order steady-state equations (see METHODS for detail). The steady-state solutions can be given finally by:


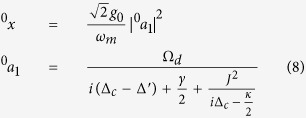


and


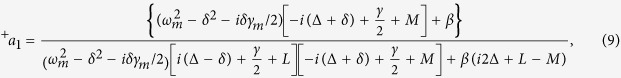


in which the undefined variables are:


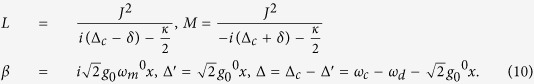


In order to obtain the OMIT behavior in this model, the output field should be solved to illustrate its response. Based on the input-output relation, the output field can be got by


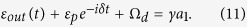


We also extend the output field *ε*_*out*_(*t*) in the form of


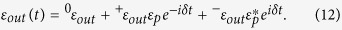


and the following relations can be achieved by substituting Eq. (12) into Eq. (11):


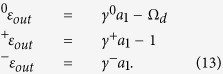


Observing OMIT phenomenon requires us to specifically focus on the response of the cavity optomechanical system to the probe field in the presence of the coupling field. In Eq. (12), the component of the output field oscillating at the probe frequency corresponds to the term ^+^*ε*_*out*_, and one can intuitively find from Eq. (13) that ^+^*ε*_*out*_ does not vary with the coupling field. Similarly to previous works, we concentrate on the behavior of ^+^*a*_1_ and define 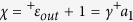 for the convenience of discussion. The relevant absorption and dispersion theories inspire us that real part and imagery part of *χ* respectively represent the behaviors of absorption and dispersion^26^.

**Figure 3:**
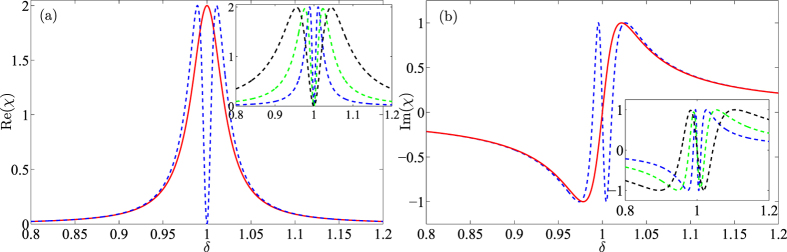
Changes of *χ* with probe field frequency. (**a**,**b**) Respectively correspond to the real and imaginary parts of *χ*. In both figures, the blue dash lines denote the *χ* in 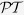-symmetric OMS (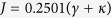) and red solid lines are *χ* in normal passive system (*J* = 0). In this simulation, the gain rate is set as weak intensity *κ*/*γ* = 10^−4^ and other parameters are the same with Fig. 2. The insets in (**a**,**b**) describe the changes of Re*χ* and Im*χ* with enlarged cavity and oscillator dissipations. Here the blue lines are of the same parameters with ones in main figures and the green lines refer to the case we amplify the cavity dissipation 2 times and oscillator dissipation 20 times (*γ* = 2/23.4 and *γ*_*m*_ = 0.38*γ*), and the black lines denote the both dissipations are amplified 4 and 40 times, respectively (*γ* = 4/23.4 and *γ*_*m*_ = 0.38*γ*).

**Figure 4:**
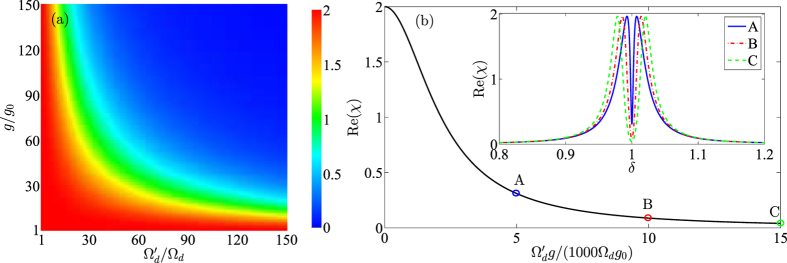
(**a**) Real part of *χ* at *δ* = *ω*_*m*_ as functions of magnified driving 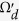 and coupling intensity *g*. (**b**) Real part of *χ* at *δ* = *ω*_*m*_ as function of 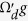. The inset in (**b**) shows the absorption spectra under 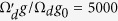 (blue), 10000 (red) and 15000 (green), respectively. The parameters here are the same with Fig. 3.

In Fig. 3(a,b), we first exhibit the enhancement effect on the OMIT phenomenon in this 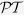-symmetric OMS (blue dashed line). The real part and imagery part of *χ* show an obvious transparent window emerges at the frequency 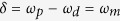. This OMIT phenomenon can still appear even in the weak driving and weak optomechanical coupling regimes, which is quite different from the OMIT in the normal passive system. When *J* = 0, the 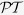-symmetric OMS returns to a normal passive system and the gain rate *κ* no longer exist and the system only has decay rates *γ* and *γ*_*m*_. Figure 3 illustrates that the absorption and dispersion properties perform like a light falling on a normal medium and there are not any transparent windows in this case since the driving and coupling are both too weak. The comparisons between blue line and red line in Fig. 3 show OMIT phenomenon is enhanced by the 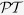-symmetric system. The insets in Fig. 3(a,b) show that such an enhancement effect in fact can be carried out in a wide range of parameters. As a criterion of enhanced OMIT, the depth of transparent window is almost unchanged even though the *γ* and *γ*_*m*_ are both enlarged. What is influenced by dissipation is the width of transparent window and it is widened significantly when *γ* and *γ*_*m*_ are increased, implying that the enhancement effect discussed in our work is a general conclusion to a certain extent, i.e., it does not require that *γ* and *γ*_*m*_ are both extremely small. It can be known from Fig. 4, that OMIT phenomenon can not be obtained in normal passive system unless there exists very strong optical driving or optomechanical coupling. In Fig. 4(a), we show that, in order to obtain OMIT, increasing the driving intensity or coupling intensity is equivalent in this system and Fig. 4(b) indicates that Ω_*d*_*g*_0_ is required to be amplified 15000 times for achieving a similar OMIT phenomenon Re(*χ*) → 0. Based on the above discussions, one can conclude that a 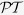-symmetric OMS can indeed enhance the OMIT phenomenon.

**Figure 5:**
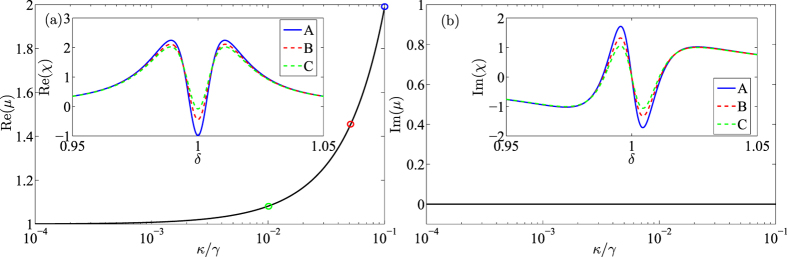
Changes of *μ* with different gain rates *κ* at the transparent frequency *δ* = *ω*_*m*_. (**a**,**b**) Respectively correspond to the real and imaginary parts of *μ*. The insets in (**a**,**b**) show the absorption and dispersion spectra under *κ* = 0.01 (green), *κ* = 0.05 (red) and *κ* = 0.1 (blue). The parameters here are the same with Fig. 3.

Further more, we introduce an optomechanically induced amplification phenomenon in this enhanced OMIT, that is, the probe field may be amplified by the active cavity if we increase its gain rate *κ*. We find that the amplification is selective, meaning that it can be realized in this system only when the probe field satisfies *ω*_*p*_ − *ω*_*d*_ = *ω*_*m*_. To describe this property, we consider the variable 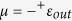 and plot it in Fig. 5. In Eq. (11), we have defined the output corresponding to the probe field is of the amplitude 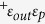. Therefore, 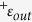 can be regard as a magnification since it is an amplitude ratio between the response and probe field. In normal OMIT phenomenon, the maximum response amplitude should be *μ* = 1 due to the conservation of energy. Physically, this case means all probe fields are exported without any dispersion. However, the existence of the active cavity allows response field to carry more energy than the probe field. In Fig. 5(a), one can observe significant amplification effect with the increasing of *κ*. In particular, the probe field will be doubled at *κ* = 0.1. It is worth noting that amplified probe (response) field is still a small quantity compared to the driving field. Thus, the dynamics analyses in Eqs (4)~(13) are still accurate. The inset in Fig. 5(a) shows that this amplification phenomenon only exists at the transparent frequency *δ* = *ω*_*m*_. Correspondingly, the dispersion spectra (see Fig. 5(b) and its inset) illustrate that there are not any dispersion effects in this case. The above two properties clarify the reason why we think this amplification is selective. Moreover, the insets in Fig. 5 show the absorption and dispersion spectra are similar with these in normal OMIT phenomena except for negative Re(*χ*). Especially, the widths of the transparency windows almost keep unchanged. Therefore, this enhanced OMIT can be directly used in some schemes of OMIT applications without changing other properties.

## Discussion

### Transformation between inverted-OMIT and OMIT

In the above section, we have already shown a transparent window (Re(*χ*) → 0) and Im(*χ*) → 0) will appear after we add an extra gain to keep the system in 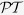
SP. A natural question is what this transparent window corresponds to, an ordinary OMIT or an inverted-OMIT. The achieved answer is that 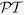 symmetry can enhance both OMIT and inverted-OMIT. The inverted-OMIT in 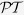-symmetric OMS has been reported in ref. 30 recently. The OMIT effect, never being observed in gain systems, can also be enhanced under certain parameters. In order to show our conclusions intuitively, we calculate the transmission rate 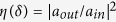, i.e., the square of amplitude ratio between the output field and input field amplitudes, to distinguish OMIT or inverted-OMIT^30^. Using the input-output relationship in Eq. (13), the transmission rate can be simplified as


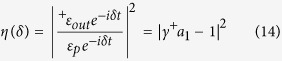


due to narrow bandwidth driving and detecting fields. Generally speaking, a typical OMIT-like transmission rate corresponds to such a one with a transparency window and two sideband dips in frequency domain. It takes on, however, a significant difference with inverted-OMIT phenomenon in which corresponding transmission rate exhibits a transmission dip and two sideband peaks^30^,^44^,^45^,^46^.

**Figure 6:**
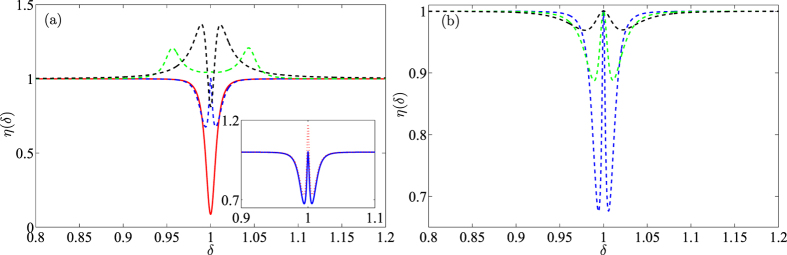
Changes of *η* with probe field frequency. (**a**) Comparison of transmission rates corresponding respectively to absorption process (red), OMIT (blue), inverted-OMIT (green) and inverted-OMIT in ref. 26. Here we set *g* = 7.4 × 10^−4^*γ*, *γ*_*m*_ = 0.38*γ* and Ω_*d*_ = 5000*γ*. The absorption, OMIT and inverted-OMIT respectively correspond to *J* = 0, 0.2501(*γ* + *κ*) and (*γ* + *κ*); *κ* = 0, 10^−4^*γ* and 0.05*γ*. The inset in (**a**) is the comparison of transmission rates corresponding to OMIT (blue, *κ* = 10^−4^*γ*) and optomechanically induced amplification (red, *κ* = 10^−2^*γ*). (**b**) Comparison of transmission rates corresponding to *γ* = 1/23 (blue), *γ* = 2/23 (green) and *γ* = 4/23 (black) with *γ*_*m*_ = 0.38*γ*. Here the other parameters are the same with the Fig. 2.

It can be known from Fig. 6(a) that under some certain parameters, one can make the system switch between transparency and inverted transparency by only adjusting the coupling intensity *J* of fiber and the gain *κ* on the auxiliary cavity. The transmission rate *η* takes on a significant decline at *δ* = *ω*_*m*_ when *J* = 0. Physically, this means that the optomechanical system corresponds to an absorption process on the probe field. When the fiber is connected (*J* ≠ 0) and its coupling is adjusted to the appropriate interaction strength, a transmission window with *η* ~ 1 appears at *δ* = *ω*_*m*_ with two sideband dips. By setting different *J* and *κ*, an inverted-OMIT phenomenon can be also observed in our system, meaning that we can achieve both slow light and fast light operations in such a system and the absorption and transparency can be of controllable flexibilities by switching the fiber. The inset in Fig. 6(a) shows that the optomechanically induced amplification phenomenon exhibits similar transmission rate curve with the OMIT phenomenon. The only difference between optomechanically induced amplification and OMIT is that *η*(*ω*) of the former will be greater than 1 because the probe field is amplified by the auxiliary cavity.

In Fig. 6(b), we show that the OMIT-like transmission rate can also appear even if the *γ* and *γ*_*m*_ are both enlarged, which is a similar conclusion with Fig. 3. In particular, the transparent window will not be affected with the increased dissipation and *η*(*ω*) ~ 1 is always satisfied. Relatively, the deep two sideband dips become shallow and the width of the transparent window will be broadened in this case.

### Physical mechanism of enhancing OMIT

Now we discuss the physical mechanism why parity-time-symmetric system can enhance OMIT by an extra gain. The underlying physics of OMIT is formally similar to that of ordinary EIT in atomic system and we know that emerging traditional EIT in Λ-type atom system requires three necessary conditions. Analogizing optomechanically induced transparency to EIT in atomic systems, Huang and Agarwal in ref. 47, discussed this relation and sketched three conditions for engendering OMIT, that is,

- Driving frequency is set in the red sideband, that is, 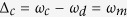.
- Optical field loss needs to be much greater than oscillator dissipation, i.e., 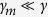.
- Steady state of oscillator displacement is not zero, i.e., 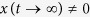.

Intuitively, conditions 2 and 3 are contradictory in a normal dissipative optomechanical system because the condition 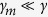 requires a large optical field decay, which will result in a decrease of the photon number in the cavity. When the photon number is reduced, the oscillator displacement will approach to equilibrium position and the condition *iii* is violated in this case. In order to solve this contradiction, strong driving is needed in previous works to ensure more photons preserved in the cavity and strong optomechanical interaction is demanded for a nonvanishing oscillator displacement *x*. That is why Fig. 4 shows the OMIT effect appears only with strong driving and optomechanical interaction.

In 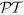-symmetric OMS, we find that the gain can increase the photon number. Under some certain parameters, however, this gain almost does not reduce the effective dissipation of the system, which ensures the condition *iii* is also well satisfied. In other words, the contradictory OMIT conditions can be well met simultaneously in 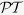-symmetric OMS even with weak optomechanical coupling and driving, and it is the reason for the positive effect of 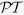-symmetric OMS on OMIT.

Now we verify the above analyses in mathematics and let us re-examine the steady state solution in Eq. (8). The oscillator displacement depends on the photon number in the cavity, which jointly affect the OMIT phenomenon via parameter *β* (see Eq. (9)). For the quantitative interpretation, we simplify *β* as


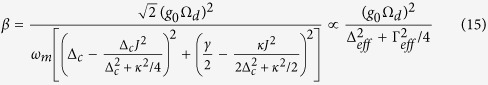


by substituting Eq. (10) into Eq. (8) and neglecting Δ′. In Eq. (15), we find that *β* depends on the product rather than independent driving and coupling intensities, and it is the reason why increasing driving intensity or coupling intensity is equivalent and hyperbolic contour appears in Fig. 4. More importantly, it can be known from Eq. (15) that the extra auxiliary cavity can provide frequency and decay corrections in the expression of *β*, that is, 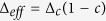 and 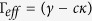, where 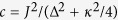. If *κ* is positive, i.e., a gain effect on the auxiliary cavity, both Δ_*eff*_ and Γ_*eff*_ will be reduced and correspondingly, *β* will be amplified because Δ_*eff*_ and Γ_*eff*_ appear in the denominator of Eq. (15).

Here we emphasize that the corrections of frequency (Δ_*eff*_) and decay (Γ_*eff*_) described above are only applicable to the discussion of *β*. In addition to the impact of *β*, the extra auxiliary cavity can also change dynamics characteristics of the system. As shown in Fig. 1(c), the cavity mode will take place a model splitting 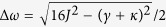 in the 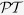-SP regime^5^,^15^. If we set *J* to be very close to the EP (*J* ~ 0.2501(*κ* + *γ*)), two splitting models will degenerate again with an effective decay *γ*_*eff*_ = *γ* − *κ* (see effective energy level diagram in Fig. 1(b)). A small *κ* allows us to ignore it, which causes the effective energy level decay is not affected by the auxiliary cavity. Therefore, the condition 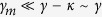 can still be well satisfied.

However, if the auxiliary cavity is also a dissipative cavity without gain, the auxiliary cavity (or other coupled quantum systems) can not always guarantee to increase *β* because Γ_*eff*_ will increase when *κ* is negative. Though *γ*_*eff*_ = *γ* + |*κ*| is increased in this case, it can not always guarantee for a positive effect on OMIT. This is the reason why our scheme is superior to other schemes which use passive quantum system to enhance OMIT^32^,^33^,^34^,^35^.

In summary, we have proposed a theoretical scheme to enhance the OMIT phenomenon in 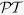-symmetric OMS. By calculating absorption (dispersion) spectrum and transmission rate, we have achieved an obvious OMIT window with experimentally accessible parameter values. In contrast to the single passive nonlinear OMS or coupled passive systems, our results illustrate that Re(*χ*) ~ 0 and Im(*χ*) ~ 0 are easy to be satisfied even though both driving and nonlinear coupling are extremely weak. This enhancement effect is due to the additional gain. Specifically, a special physical mechanism ensures that the photon number in cavity is increased without reducing effective decay, and this is the key difference between our scheme and previous works in which the gain is considered just to damage OMIT phenomenon. We have found that this scheme allows us to flexibly control absorption and transparency by switching the fiber. Moreover, the probe field can also exhibit an electromagnetically induced amplification effect with the increasing gain rate. We thus believe the scheme proposed here may provide a promising choice for the unachievable strong nonlinear coupling in quantum optical devices, which is of potential applications for coherent manipulation, slow light operation and other utilizations in QIP^48^.

## Methods

### Derivation of the absorption and dispersion spectra

As we have discussed in RESULTS, the steady-state solution of Eq. (3) can be expanded to the following form:


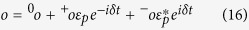


under the condition of *t* → ∞, and the relation


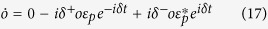


can be intuitively obtained. Substituting Eq. (17) into Eq. (3), the dynamics equations of systemic mean values become:


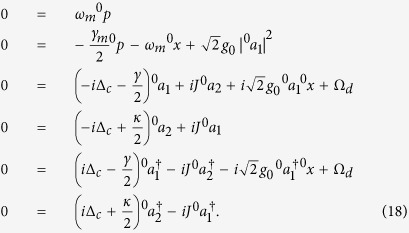


and


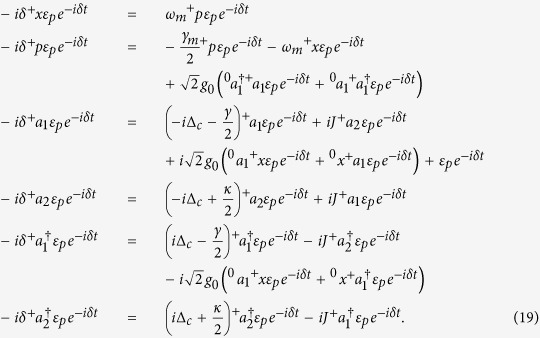


after ignoring these terms which contain 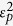, 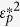 and |*ε*_*p*_|^2^. Here Eqs 18 and 19 correspond respectively to first-order and second-order steady states. Therefore, Eq. 19 can be further simplified as:


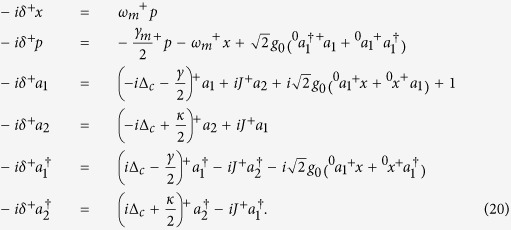


Based on Eqs 18 and 20, the steady-state solutions can be given finally by:


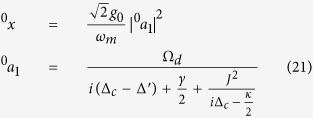


and


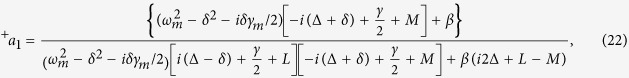


which are exactly the same with Eqs 8 and 9 in Results.

## Additional Information

**How to cite this article**: Li, W. *et al*. Parity-time-symmetry enhanced optomechanically-induced-transparency. *Sci. Rep.* **6**, 31095; doi: 10.1038/srep31095 (2016).

## References

[b1] Bender C. M.. “Making sense of non-Hermitian Hamiltonians”. Rep. Prog. Phys. 70, 947–1018 (2007).

[b2] Sendy P. . “Parity-time symmetric coupled microresonators with a dispersive gain/loss”. Opt. Express 23, 11493–11507 (2015). 25969244 10.1364/OE.23.011493

[b3] Chen Z., Wang H., Luo B. & Guo H.. “Parity-time symmetric Bragg structure in atomic vapor”. Opt. Express 22, 25120–25127 (2014). 25401544 10.1364/OE.22.025120

[b4] S. Nixon, Yi Zhu & Yang J.. “Nonlinear dynamics of wave packets in parity-time-symmetric optical lattices near the phase transition point”. Opt. Lett. 37, 4874–4876 (2012). 23202075 10.1364/OL.37.004874

[b5] JingH., ÖzdemirS. K., LüX. Y., ZhangJ., YangL. & NoriF.. “ 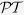 -symmetric phonon laser”. Phys. Rev. Lett. 113, 053604 (2014).2512692110.1103/PhysRevLett.113.053604

[b6] Dmitriev S. V., Sukhorukov A. A. & Kivshar Y. S.. “Binary parity-time-symmetric nonlinear lattices with balanced gain and loss”. Opt. Lett. 35, 2976–8 (2010). 20808388 10.1364/OL.35.002976

[b7] Xu X. W., Liu Y. X., Sun C. P. & Li Y.. “Mechanical PT symmetry in coupled optomechanical systems”. Phys. Rev. A 92, 013852 (2015).

[b8] Chen S. L., Chen G. Y. & Chen Y. N.. “Increase of entanglement by local PT-symmetric operations”. Phys. Rev. A 90, 054301 (2014).

[b9] Ahmed Z., Nathan J. A. & Ghosh D.. “Transparency of the complex PT-symmetric potentials for coherent injection”. Phys. Lett. A 380, 562 (2016).

[b10] Bender C. M. & Boettcher S.. “Real spectra in non-Hermitian Hamiltonians having PT symmetry”. Phys. Rev. Lett. 80, 5243 (1998).

[b11] Bender C. M., Brody D. C. & Jones H. F.. “Complex extension of quantum mechanicsy”. Phys. Rev. Lett. 89, 270401 (2002). 12513185 10.1103/PhysRevLett.89.270401

[b12] Li J., Zhan X., Ding C., Zhang D. & Wu Y.. “Enhanced nonlinear optics in coupled optical microcavities with an unbroken and broken parity-time symmetry”. Phys. Rev. A 92, 043830 (2015).

[b13] Gupta S. K. & Sarma A. K.. “Peregrine rogue wave dynamics in the continuous nonlinear Schrödinger system with parity-time symmetric Kerr nonlinearity”. Commun. Nonlinear Sci.Numer. Simulat. 36, 141–147 (2016).

[b14] Li J., Yu R. & Wu Y.. “Proposal for enhanced photon blockade in parity-time-symmetric coupled microcavities”. Phys. Rev. A 92, 053837 (2015).

[b15] LüX. Y., JingH., MaJ. Y. & WuY.. “ 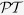 -symmetry-breaking chaos in optomechanics”. Phys. Rev. Lett. 114, 253601 (2015).2619712510.1103/PhysRevLett.114.253601

[b16] Driben R. & Malomedu B. A.. “Stability of solitons in parity-time-symmetric couplers”. Opt. Lett. 36, 4323–4325 (2011). 22089551 10.1364/OL.36.004323

[b17] NazariF. . “Optical isolation via 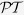 -symmetric nonlinear Fano resonances”. Opt. Express 22, 9574–9584 (2014).2478784510.1364/OE.22.009574

[b18] Peng B. . “Parity-time-symmetric whispering-gallery microcavities”. Nat. Phys. 10, 394–398 (2014).

[b19] Regensburger A. . “Parity-time synthetic photonic lattices”. Nature 488, 167–171 (2012). 22874962 10.1038/nature11298

[b20] Chang L. . “Parity-time symmetry and variable optical isolation in active-passive-coupled microresonators”. Nat. Photon. 8, 524–529 (2014).

[b21] Feng L. . “Nonreciprocal light propagation in a silicon photonic circuit”. Science 333, 729–733 (2011). 21817046 10.1126/science.1206038

[b22] Peng B. . “Loss-induced suppression and revival of lasing”. Science 346, 328–332 (2014). 25324384 10.1126/science.1258004

[b23] Liu Y. C., Xiao Y. F., Luan X. & Wong C. W.. “Dynamic dissipative cooling of a mechanical resonator in strong coupling optomechanics”. Phys. Rev. Lett. 110, 153606 (2013). 25167269 10.1103/PhysRevLett.110.153606

[b24] Zhang S. . “Ground state cooling of an optomechanical resonator assisted by a Λ-type atom”. Opt. Express 22, 28118–28131 (2014). 25402052 10.1364/OE.22.028118

[b25] Nie W., Chen A. & Lan Y.. “Cooling mechanical motion via vacuum effect of an ensemble of quantum emitters”. Opt. Express 23, 30970–84 (2015). 26698728 10.1364/OE.23.030970

[b26] Agarwal G. S. & Huang S.. “Electromagnetically induced transparency in mechanical effects of light”. Phys. Rev. A 81, 041803(R) (2010).

[b27] Ma J. . “Optomechanically induced transparency in the mechanical-mode splitting regime”. Opt. Lett. 39, 4180–4183 (2014). 25121681 10.1364/OL.39.004180

[b28] Thomas R., Kupchak C., Agarwal G. S. & Lvovsky A. I.. “Observation of electromagnetically induced transparency in evanescent fields”. Opt. Express 21, 6880–6888 (2013). 23546070 10.1364/OE.21.006880

[b29] Abi-Salloum T. Y.. “Electromagnetically induced transparency and Autler-Townes splitting: Two similar but distinct phenomena in two categories of three-level atomic systems”. Phys. Rev. A 81, 053836 (2010).

[b30] Jing H. . “Optomechanically-induced transparency in parity-time-symmetric microresonators”. Sci. Rep. 5, 9663 (2015). 26169253 10.1038/srep09663 PMC4500988

[b31] Aspelmeyer M., Kippenberg T. J. & Marquardt F.. “Cavity optomechanics”. Rev. Mod. Phys. 86, 1391–1452 (2014).

[b32] Xiao Y., Yu Y. F. & Zhang Z. M.. “Controllable optomechanically induced transparency and ponderomotive squeezing in an optomechanical system assisted by an atomic ensemble”. Opt. Express 22, 17979–17989 (2014). 25089417 10.1364/OE.22.017979

[b33] Turek Y., Li Y. & Sun C. P.. “Electromagnetically-induced-transparency–like phenomenon with two atomic ensembles in a cavity”. Phys. Rev. A 88, 053827 (2013).

[b34] Han Y., Cheng J. & Zhou L.. “Electromagnetically induced transparency in a cavity optomechanical system with an atomic medium”. J. Phys. B. At. Mol. Opt. Phys. 44, 165505–165509 (2011).

[b35] Chang Y., Shi T., Liu Y. X, Sun C. P. & Nori F.. “Multistability of electromagnetically induced transparency in atom-assisted optomechanical cavities”. Phys. Rev. A 83, 063826 (2011).

[b36] Farace A. & Giovannetti V.. “Enhancing quantum effects via periodic modulations in optomechanical systems”. Phys. Rev. A 86, 013820 (2012).

[b37] Liu Z., Lai Y. C. & Matas M. A.. “Universal scaling of Lyapunov exponents in coupled chaotic oscillators”. Phys. Rev. E 67, 045203(R) (2003). 10.1103/PhysRevE.67.045203 12786425

[b38] Lü L. . “The signal synchronization transmission among uncertain discrete networks with different nodes”. Nonlinear Dyn. 81, 801–809 (2015).

[b39] Lü L. . “Spatiotemporal chaos synchronization of an uncertain network based on sliding mode control”. Chin. Phys. B 21, 100507–5 (2012).

[b40] Li W. L., Li C. & Song H. S.. “Quantum parameter identification for a chaotic atom ensemble system”. Phys. Lett. A 380, 672–677 (2016).

[b41] Eichenfield M. . “A picogram- and nanometre-scale photonic-crystal optomechanical cavity”. Nat. Lett. 459, 550–555 (2009). 10.1038/nature08061 19489118

[b42] Eichenfield M. . “Optomechanical crystals”. Nat. Lett. 462, 78–82 (2009). 10.1038/nature08524 19838165

[b43] Wang H., Sun H. C., Zhang J. & Liu Y. X.. “Transparency and amplification in a hybrid system of the mechanical resonator and circuit QED”. Sci. China-Phys. Mech. Astron. 55, 2264–2272 (2012).

[b44] Oishi T. & Tomita M.. “Inverted coupled-resonator-induced transparency”. Phys. Rev. A 88, 013813 (2013).

[b45] Kou J. . “EIT-assisted large cross-Kerr nonlinearity in a four-level inverted-Y atomic system”. J. Opt. Soc. Am. B 27, 2035–2039 (2010).

[b46] Ma P. C., Zhang J. Q., Feng M. & Zhang Z. M.. “Bidirectional and tunable single-photons multi-channel quantum router between microwave and optical light”. *arXiv*:1410.4371v1 (2014).

[b47] Huang S. & Agarwal G. S.. “Electromagnetically induced transparency from two-phonon processes in quadratically coupled membranes”. Phys. Rev. A 83, 023823 (2011).

[b48] Zhang J., Hernandez G. & Zhu Y.. “Copropagating superluminal and slow light manifested by electromagnetically assisted nonlinear optical processes”. Opt. Lett. 31, 2598–2600 (2006). 16902631 10.1364/ol.31.002598

